# Emerging role of transient receptor potential (TRP) ion channels in cardiac fibroblast pathophysiology

**DOI:** 10.3389/fphys.2022.968393

**Published:** 2022-10-06

**Authors:** Asfree Gwanyanya, Kanigula Mubagwa

**Affiliations:** ^1^ Department of Human Biology, University of Cape Town, Cape Town, South Africa; ^2^ Department of Cardiovascular Sciences, K U Leuven, Leuven, Belgium; ^3^ Department of Basic Sciences, Faculty of Medicine, Université Catholique de Bukavu, Bukavu, Democratic Republic of Congo

**Keywords:** transient receptor potential, ion channel, fibroblast, myofibroblast, cardiac

## Abstract

Cardiac fibroblasts make up a major proportion of non-excitable cells in the heart and contribute to the cardiac structural integrity and maintenance of the extracellular matrix. During myocardial injury, fibroblasts can be activated to trans-differentiate into myofibroblasts, which secrete extracellular matrix components as part of healing, but may also induce cardiac fibrosis and pathological cardiac structural and electrical remodeling. The mechanisms regulating such cellular processes still require clarification, but the identification of transient receptor potential (TRP) channels in cardiac fibroblasts could provide further insights into the fibroblast-related pathophysiology. TRP proteins belong to a diverse superfamily, with subgroups such as the canonical (TRPC), vanilloid (TRPV), melastatin (TRPM), ankyrin (TRPA), polycystin (TRPP), and mucolipin (TRPML). Several TRP proteins form non-selective channels that are permeable to cations like Na^+^ and Ca^2+^ and are activated by various chemical and physical stimuli. This review highlights the role of TRP channels in cardiac fibroblasts and the possible underlying signaling mechanisms. Changes in the expression or activity of TRPs such as TRPCs, TRPVs, TRPMs, and TRPA channels modulate cardiac fibroblasts and myofibroblasts, especially under pathological conditions. Such TRPs contribute to cardiac fibroblast proliferation and differentiation as well as to disease conditions such as cardiac fibrosis, atrial fibrillation, and fibroblast metal toxicity. Thus, TRP channels in fibroblasts represent potential drug targets in cardiac disease.

## 1 Introduction: Fibroblasts as important myocardial cellular components

Traditionally, the understanding of heart function at the cellular level has focused on contractile cells (i.e., atrial and ventricular myocytes) as well as pacemaker and conduction cells. Hence, cardiac diseases such as heart failure have been understood as being mainly due to a dysfunction of these cells: loss of contractile function to account for pump failure, and abnormalities of electrical impulse initiation and conduction to account for arrhythmias. With growing interest in conditions such as heart failure with preserved ejection fraction where there is no apparent loss of cardiomyocytes or of conduction cells, there has also been an increase in the attention given to other cardiac cell types and to the extracellular matrix as well as to their alterations under disease conditions (Gevaert et al., 2019). The other cell types within cardiac tissues include endothelial cells (in the endocardium and in vessels), smooth muscle cells (in the coronary vessels), and fibroblasts (in the interstitium). The latter are reported to represent at least 27% up to nearly two-thirds of the total adult cardiac cell population (Banerjee et al., 2007; Ongstad and Kohl, 2016), and the cell proportion may change in disease conditions (Moore-Morris et al., 2015). In some extreme conditions, increased proliferation of fibroblasts and increased synthesis and deposition of modified extracellular matrix proteins may develop, leading to cardiac fibrosis, which is increasingly recognized as being a hallmark of heart failure (Schimmel et al., 2022).

Cardiac fibroblasts are non-excitable cells that play key roles in the normal myocardium as well as during myocardial healing and in cardiac disease (Soliman and Rossi, 2020). Individually, they can be identified as spindle-shaped flat cells that express biomarkers such as discoidin domain receptor 2 (DDR2) and vimentin (Tarbit et al., 2019). The origin of cardiac fibroblasts is heterogeneous: besides the resident fibroblasts which derive from mesenchymal trans-differentiation of epicardial or endocardial cells especially during embryonic development, others derive from pericytes surrounding capillaries or from pluripotent (hematopoietic, fibrocyte) progenitor cells (Aujla and Kassiri, 2021). Functionally, cardiac fibroblasts synthesize and secrete components of the extracellular matrix (ECM) surrounding them, including collagen fibres (mainly type I but also type III) and ground substances containing mucopolysaccharides, proteoglycans, and multi-adhesive glycoproteins (Deleon-Pennell et al., 2020). The secretion of ECM components is kept in balance *via* the opposite actions of proteolytic enzymes such as proteases like metalloproteinases and of anti-proteases (Umbarkar et al., 2021). The ECM components and the fibroblast network together contribute to the structural and mechanical integrity of the heart, which ultimately determines the compliance and modulates the contraction/relaxation characteristics of the myocardium. In addition to their role in determining the ECM, the fibroblast-cardiomyocyte coupling involves not only structural connections *via* gap junctions that influence the cell membrane electrical properties or *via* desmosomes, but also involves chemically mediated connections *via* paracrine secretions. These structural and functional interactions of fibroblasts with cardiomyocytes (Hall et al., 2021) play a role in cardiac electrical conduction and rhythmicity (Gaudesius et al., 2003; Kohl et al., 2005; Miragoli et al., 2006; Sridhar et al., 2017).

Being part of the myocardium, cardiac fibroblasts exist and function in a demanding micro-environment with continuous episodic mechanical stress and high metabolic requirements, and therefore, are subjected to a variety of physical and chemical stimuli. The role of fibroblasts in ECM homeostasis is attributed to their ability to respond to increased (mechanical and other types of) stress. This response consists of increased cell proliferation and ECM protein synthesis. During myocardial injury, cardiac fibroblasts can be triggered to migrate to the site of injury by chemotactic factors and pro-inflammatory cytokines as well as be activated to trans-differentiate into myofibroblasts. The latter are secretory and contractile cells with an even greater capacity for fibrinogenesis and proliferation, resulting in the remodeling of the ECM and the development of fibrosis and the formation of fibrotic scars (Davis and Molkentin, 2014). Myofibroblasts can be identified by their fibroblast-endothelial cell-like features such as the expression of α-smooth muscle actin (α-SMA) (Tarbit et al., 2019), and are not expected to be present in the myocardium after healing, unless there is persistent stress or fibrinogenesis dysregulation that leads to chronic cardiac fibrosis. Cardiac fibrosis develops following excessive activation of fibroblasts by various mechanical and chemical signaling factors (see below).

The mechanisms through which cardiac fibroblasts and myofibroblasts are activated and regulated have been detailed in comprehensive reviews (Weber et al., 2013; Frangogiannis, 2019; Tarbit et al., 2019; Umbarkar et al., 2021), which highlight that the precise molecular pathways involved are still not fully understood. Broadly, the triggers for fibroblast proliferation and differentiation include stress-related factors derived from mechanical forces or from the activation of neurohumoral pathways or from inflammatory processes. The profibrotic neurohumoral factors include aldosterone (Stockand and Meszaros, 2003) and angiotensin II, produced locally *via* the release of renin and angiotensin-converting enzyme by injured cardiomyocytes, infiltrating macrophages, and other fibroblasts (Weber et al., 1997; Sun et al., 2001). In addition, there is release of profibrotic endothelin-1 by the injured myocardium (Katwa, 2003) and adrenergic neurotransmitters from cardiac sympathetic overdrive (Levick et al., 2010). The profibrotic by-products of inflammation include cytokines like tumour necrosis factor alpha (TNF-α) and interleukins (Sun et al., 2007; Fix et al., 2011; Schafer et al., 2017) as well as cytokine-like growth factors such as the transforming growth factor beta 1 (TGF-β1) (Du et al., 2017; Gao et al., 2019; Vallee and Lecarpentier, 2019), produced by macrophages and other interstitial immune cells. TGF-β1, in particular, is a key profibrotic trigger that also cross-talks with other modulators of fibrosis such as angiotensin II, Wnt proteins and microRNAs (Rosenkranz, 2004; Yousefi et al., 2020; Dzialo et al., 2021). Antifibrotic humoral factors include connective tissue growth factor, eventually produced by cardiomyocytes (Aranguiz et al., 2021) and natriuretic peptides (Kapoun et al., 2004).

Intracellularly, TGF-β1 receptor-mediated effects occur *via* either Smad protein-mediated (canonical) pathways or Smad-independent non-canonical pathways (Hanna et al., 2021). Alternatively, oxidative stress can be produced from reactive oxygen species (ROS)-generating NADPH oxidases in response to pro-inflammatory cytokines or indirectly *via* Ca^2+^ overload (Chan et al., 2013). In turn, the oxidative stress stimulates ROS-sensitive kinases such as mitogen-activated protein kinases (MAPKs) or activates the small GTP-binding protein RhoA and Rho-associated coiled-coil containing kinases (ROCKs) (Jatho et al., 2015). The downstream signaling molecules of TGF-β1, MAPK, or RhoA can interact with regulators of gene expression such as microRNAs (Chandy, 2019; Feng et al., 2022), or with the proposed antifibrotic proteins [e.g., sirtuins (Cappetta et al., 2016; Maity et al., 2020)], or activate the secretion of Wnt proteins, which induce profibrotic effects *via* either β-catenin or β-catenin-independent pathway (Yousefi et al., 2020; Dzialo et al., 2021). Finally, intracellular Ca^2+^ oscillations play an important role in fibroblast action and myofibroblast contraction as well as trigger oxidative stress or downstream Ca^2+^-dependent pathways involved in pathological cardiac remodeling (Liu et al., 2012).

Increased intracellular Ca^2+^ levels seem to play a key role, and result in the activation of Ca^2+^-dependent proteases (e.g., calcineurin) and the subsequent translocation of the nuclear factor of activated T cells (NFAT) to the nucleus (Chen et al., 2012). However, fibroblasts lack voltage-gated Ca^2+^ channels and depend on alternative Ca^2+^ influx pathways. The potential pathways for Ca^2+^ entry in fibroblasts include those in exchange with Na^+^ extrusion *via* the Na^+^-Ca^2+^ exchanger operating in reverse-mode (Kamimura et al., 2012) or in exchange with Mg^2+^ extrusion *via* the Ca^2+^-Mg^2+^ exchanger SLC41A1 (Yu N. et al., 2014). Ca^2+^ entry *via* members of the Ca^2+^ release-activated or Orai family of channels has also been proposed (Camacho Londono et al., 2020). The other potential Ca^2+^-entry pathways in fibroblasts are transient receptor potential (TRP) ion channels, which are generally Ca^2+^-permeable. These channels act as biosensors of different physical and chemical stimuli and as mediators of extracellular and intracellular signaling in several types of cells, including myocardial cells (Hof et al., 2019; Inoue et al., 2019). As such, understanding the role of TRP channels may provide further insights into fibroblast (patho)physiology. In the present review, we provide an overview of TRP channels and outline their role in cardiac fibroblasts, including the possible underlying signaling mechanisms.

## 2 Overview of TRP channels

TRP channels are a large family of proteins classified according to their structural sequence homology. Historically, the trp gene and TRP proteins were identified in a mutant *drosophila melanogaster* with a defect in the Ca^2+^ influx component of the visual transduction pathway (Hardie and Minke, 1992). Since then, almost 30 TRPs have been identified and classified into subgroups (Nilius and Owsianik, 2011; Yue and Xu, 2021): canonical (TRPC), vanilloid (TRPV), melastatin (TRPM), ankyrin (TRPA), polycystin (TRPP), mucolipin (TRPML), as well as the *no mechanoreceptor potential* (TRPN). Many TRP channels are present in the heart [see (Freichel et al., 2017)], in which they may participate in the regulation of normal function or in pathophysiological processes (Hof et al., 2019; Inoue et al., 2019).

TRP channels have special structural and functional properties that are potentially relevant to cardiac fibroblast physiology and pathophysiology. The majority of TRP proteins form relatively non-selective channels, permeable to various monovalent cations (e.g., Na^+^) and divalent cations (e.g., Ca^2+^ and Mg^2+^), except for TRPM4 and TRPM5, which are monovalent cation-selective (Guinamard et al., 2011) and TRPV5 and TRPV6, which are relatively Ca^2+^ selective [see (Peng et al., 2018)]. Being non-excitable cells, cardiac fibroblasts lack voltage-gated ion channels, and therefore TRPs in fibroblasts may provide influx pathways for essential cations such as Ca^2+^, Mg ^2+^, Na^+^, and trace elements. Despite the lack of large ion fluxes *via* voltage-dependent channels, influxes of cations *via* TRP channels may have pronounced effects on cytosolic cation concentrations in fibroblasts given that their surface area-to-volume ratio (10–20 μm^−1^) is much larger than that of the other cardiac cells such as cardiomyocytes (4 μm^−1^) as calculated using data obtained in previous studies (Squier et al., 1990; Satoh et al., 1996; Revell et al., 2006). As such, TRPs in fibroblasts may also mediate cellular cation overload (e.g., Ca^2+^ overload) or serve as influx pathways for toxic cations.

Consistent with their wide cellular expression patterns, TRPs are activated or modulated by a variety of physical and chemical stimuli, many of which are present in the environment of cardiac fibroblasts. Regarding physical stimuli, mechanical stress can modulate TRPs such as TRPC1, TRPC3, TRPC5, TRPC6, TRPV1, TRPV2, TRPV4, TRPM3, TRPM4, TRPM7, TRPA1, and TRPP2 (Inoue et al., 2009; Liu and Montell, 2015), although the mechano-sensitivity of several of these TRPs has also been questioned (Nikolaev et al., 2019). Temperature changes can also modulate TRPV1 and TRPV2 (noxious heat) or TRPV3, TRPV4, TRPM4 and TRPM5 (warmth), or TRPM8 and TRPA1 (cold) (Huang et al., 2006). For chemical stimuli, several TRPC channels are activated by membrane phospholipid breakdown by-products such as diacylglycerol following ligand binding to G-protein-coupled receptors (Albert, 2011), whereas TRPM4 and TRPM5 channels are activated by intracellular Ca^2+^ (Guinamard et al., 2011), and TRPM6 and TRPM7 are inhibited by intracellular Mg^2+^ (Nadler et al., 2001; Voets et al., 2004). In addition, TRPCs such as TRPC1 and TRPC3 have been linked to Ca^2+^ store depletion-mediated Ca^2+^ entry (Cheng et al., 2013). Besides these broad categorizations, several TRPs show polymodal activation as well as modulation by other factors such as pH, membrane phospholipids, nucleotides, osmolarity, and transmembrane voltage (Nilius and Owsianik, 2011). Given that different types of TRP channels can be expressed in the same cell, and that cells such as cardiac fibroblasts are exposed to a variety of stimuli, the TRPs provide cells with mechanisms for polymodal bio-sensation and signal transduction.

Some TRP channels have unique structural components that may be biologically active or act as sites of protein-protein interactions among TRPs or between TRPs and other cellular components [see (Gaudet, 2007)]. The C-termini of some TRP transmembrane polypeptides may contain specific structures such as the ADP ribose-binding motif in TRPM2, kinases in TRPM6 and TRPM7, amino acid binding (PDZ) motifs in TRPC4 and TRPC5, coiled-coil domain in TRPV1, TRPM4, and TRPM8, and the calmodulin binding sites in TRPC3 and TRPC4 and *drosophila* TRP and TRPL. On the other hand, the N-termini of TRPV, TRPA, and TRPN contain ankyrin-binding motifs. Generally, the biological significance of such TRP sub-components remains an area of active research.

## 3 TRP channel expression and roles in cardiac fibroblasts

### 3.1 TRPC channels

#### 3.1.1 TRPC3

TRPC3 is one of the G-protein-coupled receptor-activated Ca^2+^-permeable TRPC channels that has been studied in human isolated atrial fibroblasts (Harada et al., 2012; Han et al., 2020) or cultured ventricular fibroblasts (Saliba et al., 2019) as well as in wild-type or TRPC3 knockout mice (Numaga-Tomita et al., 2016; Han et al., 2020), rat freshly isolated or cultured fibroblasts (Numaga-Tomita et al., 2016; He et al., 2019; Saliba et al., 2019), and canine cultured atrial fibroblasts (Harada et al., 2012). The changes in TRPC3 mRNA or protein expression as well as channel activity or current observed in these studies suggest that TRPC3 contributes to fibroblast proliferation (Harada et al., 2012; He et al., 2019), fibroblast migration (He et al., 2019), and myofibroblast differentiation as well as fibrinogenesis (Harada et al., 2012; Numaga-Tomita et al., 2016; He et al., 2019; Saliba et al., 2019; Han et al., 2020). In addition, the baseline level of expression of TRPC3 has been shown to be higher in cultured fibroblasts from the rat left atrium compared to the right atrium as was evidenced by the greater TRPC3 protein expression and larger TRPC3-like currents in left atrial fibroblasts (Chung et al., 2021), a result that is consistent with the proposed greater occurrence of fibrosis in the left atrium than in the right atrium. The TRPC3-mediated profibrotic effects are linked to cardiac disease conditions such as atrial fibrillation (Harada et al., 2012; Han et al., 2020), hypertension (He et al., 2019), and pressure overload (Numaga-Tomita et al., 2016), where the disease conditions directly or indirectly upregulate the expression of TRPC3 or activate the channels.

At the cellular level, TRPC3-mediated fibrosis can occur in response to pathological stress due to the stimulation of fibroblasts with the profibrotic cytokine TGF-β1, acting *via* its canonical pathway signaling proteins Smad2/3 (He et al., 2019; Han et al., 2020) or *via* the non-canonical pathway involving the extracellular signal-regulated kinases 1 and 2 (ERK1/2) (Harada et al., 2012). Furthermore, TRPC3-mediated fibrosis can be stimulated by G-protein-coupled receptor agonists like angiotensin II (Harada et al., 2012; Saliba et al., 2019; Han et al., 2020) and homocysteine (Han et al., 2020). For its other intracellular profibrotic effects on cardiac fibroblasts, TRPC3 may act *via* intracellular Ca^2+^ or *via* channel interactions with either profibrotic or antifibrotic molecules. The activation of TRPC3 channels in fibroblasts mediates Ca^2+^ influx (Harada et al., 2012; Saliba et al., 2019), which in turn may activate the Ca^2+^-dependent NFAT (Saliba et al., 2019), a transcription factor involved in cardiac pathological remodelling and cardiac hypertrophy (Chen et al., 2012; Liu et al., 2012). TRPC3 has also been shown to induce fibrosis through activating Rho-GTPase (Numaga-Tomita et al., 2016), or *via* interactions with the membrane bound NADPH oxidase (NOX-2) in ROS-induced fibrosis (Numaga-Tomita et al., 2017), or *via* the modulation of the proposed antifibrotic factor sirtuin 1 (Cappetta et al., 2016; Han et al., 2020).

#### 3.1.2 TRPC6

TRPC6 is the other G-protein-coupled receptor-activated TRPC channel that has also been studied in fibroblasts of cultured human ventricular tissue (Ikeda et al., 2013; Kapur et al., 2014), mouse models of TRPC6 knockout or right ventricular pressure-overload (Davis et al., 2012; Kapur et al., 2014), and cultured rat ventricular tissue (Nishida et al., 2007). In such studies, the TRPC6 changes in channel activity or expression of mRNA or protein in cardiac fibroblasts suggest that TRPC6 has mixed effects on fibroblast activity. On the one hand, TRPC6 appears to be necessary for cardiac scar formation post myocardial infarction, since TRPC6 null-mutant mice demonstrate a higher incidence of cardiac rupture, a lower recovery of function, a smaller wall scar and greater ventricular dilatation (Davis et al., 2012)*.* TRPC6 promotes cardiac fibroblast proliferation, myofibroblast differentiation, and fibroblast Ca^2+^ influx in response to TGF-β1 stimulation (Davis et al., 2012; Ikeda et al., 2013; Kapur et al., 2014) *via* the Smad3 pathway as well as *via* the ERK1/2 pathway (Davis et al., 2012; Kapur et al., 2014). In addition, TRPC6 in cardiac fibroblasts is proposed to mediate the Ca^2+^ influx that could be induced by 1-oleoyl-2-acetyl-sn-glycerol (OAG) (Ikeda et al., 2013), an analogue of diacylglycerol, which is a by-product of the membrane lipid phosphatidylinositol-4,5-bisphosphate (PIP_2_) breakdown. In turn, the Ca^2+^ influx in fibroblasts can modulate proliferation or alter Ca^2+^-dependent cardiac remodeling molecules such as calcineurin and NFAT (Kapur et al., 2014). In ventricular fibroblasts, TGF-β1 activation upregulated the expression of both TRPC6 and the Ca^2+^-dependent protein calcineurin, whereas TPRC6 silencing decreased the TGF-β1-mediated upregulation of calcineurin (Kapur et al., 2014). Similar TGF-β1-mediated profibrotic effects of TRPC6 involving calcineurin/NFAT have also been reported in pulmonary fibrosis (Kapur et al., 2014; Hofmann et al., 2017). On the other hand, TRPC6 suppresses the endothelin-1 induced activation of fibroblasts that is mediated by Gα_12/13_ proteins and ROS (Nishida et al., 2007). In that study, the over-expression of TRPC6 and NFAT decreased endothelin-1 induced myofibroblast formation, with opposite effects observed in TRPC6-silenced and NFAT-inhibited fibroblasts (Nishida et al., 2007). It could therefore be that TRPC6 may mediate either profibrotic or antifibrotic effects, depending on the nature of the stimulus on the cardiac fibroblasts.

#### 3.1.3 Other TRPCs

It is unclear which specific type of TRPC mediates the ADP-induced purinergic receptor (P2Y)-mediated Ca^2+^ influx, leading to myofibroblast growth and fibrinogenesis in rat ventricular cells (Certal et al., 2017). Nonetheless, such a limitation may not be surprizing in cases where pharmacological probes are used to identify TRPCs, given the TRPCs’ high structural sequence homology (especially among TRPC3, TRPC6, and TRPC7), the possible formation of hetero-multimers (e.g., TRPC1 with TRPC4 or TRPC5), the lack of specific blockers, and the multi-modal forms of channel activation, including G-protein-coupled receptor modulation and possibly Ca^2+^ store-operated activation [see review (Albert, 2011)]. In the study by Certal et al. (Certal et al., 2017), the ADP effects mimicked by the diacylglycerol analogue OAG could potentially be attributed to several OAG-sensitive TRPCs such as C1, C3, C6, and C7. In addition, the channel block by chemicals such as 2-APB and flufenamic acid can also occur in several TRPCs, some TRPMs, and non-TRP channels. However, Certal et al. (2017) ruled out the involvement of a non-TRP channel that is also blocked by 2-APB, the Ca^2+^ store-operated channel called Ca^2+^ release-activated Ca^2+^ (CRAC) channel by using specific CRAC channel inhibitors, thereby leaving TRPCs as the likely mediators of the ADP-induced profibrotic effects.

As for TRPC7 specifically, the conformational changes observed in the TRPC7 channel in response to angiotensin II stimulation in rat fibroblasts imply a role for the channel in fibrosis (Petigny et al., 2022). However, the TRPC7 protein is not detectable in human fibroblasts (Ikeda et al., 2013) and its mRNA is not altered in response to profibrotic endothelin-1 stimulation in rat neonatal fibroblasts (Nishida et al., 2007), hence making questionable the role of TRPC7. Similarly, the involvement of TRPC1 in fibroblasts also remains uncertain. The expression of TRPC1 (as detected using RT-PCR, western blot, immunochemistry and functional measurements) in human cardiac fibroblasts was upregulated by TGF-β1 stimulation (Ikeda et al., 2013), but the role of the channel in fibroblasts was not further explored. In addition, TRPC1/C4 proteins were shown to be required to mediate the Ca^2+^ influx implicated in pressure overload-induced cardiac interstitial fibrosis in mice (Camacho Londono et al., 2015). In contrast, there was no detectable TRPC1 expression measured at mRNA and protein levels in rat cardiac fibroblasts, even under conditions in which the channel could be detected in other cells found in the heart such as cardiomyocytes, endothelial cells, and smooth muscle cells (Huang et al., 2009). Finally, TRPC1, TRPC3, and TRPC4 were shown to have no role in angiotensin II induced Ca^2+^ influx in mouse fibroblasts although their mRNA could be detected (Camacho Londono et al., 2020).

### 3.2 TRPM channels

#### 3.2.1 TRPM2

The TRPM2 channel is generally considered as a metabolic sensor in reference to the presence of its cytoplasmic ADP ribose-binding domain. In cultured rat cardiac fibroblasts, hypoxia was shown to upregulate TRPM2 mRNA expression and to induce a TRPM2-like current that could be enhanced by intracellular ADP ribose or prevented by TRPM2 RNA interference (Takahashi et al., 2012). The results of that study suggest a role of TRPM2 as a hypoxic sensor in fibroblasts, but the implications on the fibroblast integrity or function during hypoxia remain unclear.

#### 3.2.2 TRPM4

TRPM4, a Ca^2+^-activated, monovalent cation-permeable channel, is expressed in cardiac fibroblasts, in which it mediates profibrotic effects. The expression of TRPM4 protein and the magnitude of TRPM4 current have been shown to be upregulated in either freshly isolated or cultured human ventricular fibroblasts of heart failure patients (Feng et al., 2021). In addition, the TRPM4 current was upregulated upon stimulation with the profibrotic cytokine TGF-β1 *in vitro* (Feng et al., 2021). Given the pro-hypertrophy and pro-arrhythmic roles of TRPM4 when it is expressed in cardiomyocytes (Guinamard et al., 2006; Liu et al., 2010), the profibrotic effect in cardiac fibroblasts makes TRPM4 a key disease substrate in myocardial dysfunction and arrhythmogenesis.

#### 3.2.3 TRPM7

TRPM7 is regulated by intracellular Mg^2+^ and nucleotides, and possesses both channel and kinase functions (Nadler et al., 2001; Runnels et al., 2001) (hence its designation as a chanzyme). The channel has been studied in freshly isolated and cultured human atrial fibroblasts (Du et al., 2010), mouse cultured fibroblasts (Li et al., 2008; Jia T. et al., 2021), and rat isolated and cultured fibroblasts (Yu N. et al., 2014; Guo et al., 2014; Zhou et al., 2015; Li et al., 2017; Lu et al., 2017; Zhong H. et al., 2018; Wu et al., 2018; Jia X. et al., 2021). The evidence from changes in TRPM7 channel activity and the expression of protein or mRNA in cardiac fibroblasts indicate that, in general, the channel promotes fibroblast proliferation and fibroblast-myofibroblast trans-differentiation (Du et al., 2010; Guo et al., 2014; Li et al., 2017; Lu et al., 2017; Wu et al., 2018) as well as fibrinogenesis (Du et al., 2010; Guo et al., 2014; Zhou et al., 2015; Li et al., 2017; Lu et al., 2017; Zhong H. et al., 2018; Wu et al., 2018). Such TRPM7-mediated profibrotic effects occur in cardiac disease conditions such as myocardial infarction (Li et al., 2008), atrial fibrillation (Du et al., 2010), and sick sinus syndrome (Zhong H. et al., 2018) as well as following receptor-mediated cardiac stimulation with agonists like angiotensin II (Yu Y. et al., 2014; Zhou et al., 2015; Li et al., 2017; Zhong H. et al., 2018) and isoprenaline (Li et al., 2017; Wu et al., 2018). In addition, the TRPM7-linked fibrinogenesis is induced or enhanced by metabolic stress factors such as hypoxia (Li et al., 2017), acidosis (Li et al., 2008), and hydrogen peroxide (Guo et al., 2014).

The profibrotic effects of TRPM7 in fibroblasts are proposed to be mediated *via* Ca^2+^ influx through the channels (Li et al., 2008; Du et al., 2010; Guo et al., 2014; Jia T. et al., 2021), which may be enhanced by the upregulation of TRPM7 protein expression under various pathological conditions (Li et al., 2008; Du et al., 2010; Yu Y. et al., 2014; Zhou et al., 2015; Wu et al., 2018). As such, the blockade of TRPM7 channels attenuates fibrosis (Yu Y. et al., 2014; Jia T. et al., 2021). However, an enhanced Ca^2+^ influx could occur even in the absence of upregulated TRPM7 expression, since enhanced Ca^2+^ influx can occur upon channel modulation by extracellular acidosis (Macianskiene et al., 2017; Jia T. et al., 2021). In addition to Ca^2+^ influx through TRPM7 channel, Mg^2+^ influx has also been shown to be required for angiotensin II induced fibrinogenesis in rat ventricular fibroblasts (Yu Y. et al., 2014), but the mode of action of Mg^2+^ is unclear since the Mg^2+^ extrusion by the Ca^2+^-Mg^2+^ exchanger, rather than its influx, is implicated in fibrinogenesis (Yu N. et al., 2014).

The cell signaling molecules involved in TRPM7-mediated fibroblast activation include the cytokine TGF-β1 (Li et al., 2008; Du et al., 2010; Guo et al., 2014; Jia T. et al., 2021) acting *via* the Smad pathway (Zhong H. et al., 2018) or *via* the ERK1/2 pathway (Guo et al., 2014). TRPM7 also acts *via* its interactions with potential antifibrotic molecules like micro-RNA-135a (Wu et al., 2018), but such mechanisms still require further clarifications. Furthermore, in one study, the angiotensin II induced upregulation of the TRPM7 protein expression and of the TRPM7 current as well as the fibrinogenesis were shown be short-lived, despite continued stimulation (Zhou et al., 2015), indicating the possible existence of other counter-regulatory mechanisms.

In contrast to profibrotic effects of TRPM7 when the channel is expressed in cardiac fibroblasts, TRPM7 in cardiac macrophages suppressed cardiac fibroblast activity during inflammation (Rios et al., 2020). With TRPM7 being a chanzyme, the presence of the TRPM7 kinase in macrophages was shown to be required to prevent the macrophage stimulation of fibroblasts during inflammation, a process that was regulated by the TRPM7 inhibitor Mg^2+^ (Rios et al., 2020). This finding by Rios et al. (Rios et al., 2020) suggests a role for the TRPM7 kinase in cell-cell signaling and that the presence of TRPM7 in other heart cells may, in turn, alter fibroblastic activity. Therefore, given that TRPM7 is also present in other cells found in the heart like atrial (Zhang et al., 2012; Andriule et al., 2021) and ventricular myocytes (Gwanyanya et al., 2021; Alatrag et al., 2022), it will be worth investigating whether the TRPM7 in those cells could modulate cardiac fibroblasts under certain conditions.

### 3.3 TRPV channels

#### 3.3.1 TRPV1

The capsaicin receptor TRPV1 in cardiac fibroblasts has been characterized in wild-type mice (Horton et al., 2013) and TRPV1 knockout mice (Huang et al., 2010; Buckley and Stokes, 2011; Wang et al., 2014; Wang et al., 2016; Zhong B. et al., 2018). The channel has been linked with antifibrotic cardiac effects in most studies, but also with profibrosis in other studies. The TRPV1 agonist capsaicin has been shown to decrease the angiotensin II induced fibroblast proliferation as well as to attenuate pressure-overload induced cardiac fibrosis, but not in TRPV1 knock-out mice (Wang et al., 2014). In addition, TRPV1 is cardio-protective against ischemia, since TRPV1 null-mutant mice are more susceptible to myocardial infarction-induced, TGF-β1/Smad2-mediated myofibroblast activation and fibrinogenesis (Huang et al., 2010). Similarly, under conditions where TRPV1 prevented hypertrophy, it also decreased fibrosis (Horton et al., 2013; Zhong B. et al., 2018). In the same way, the over-expression of TRPV1 in mice is protective against isoprenaline-induced cardiac fibroblast proliferation and collagen deposition, effects that were mediated by Ca^2+^ influx and the endothelial nitric oxide synthase [eNOS (Wang et al., 2016)]. Given that the Ca^2+^ influx through other TRPV channels (TRPV3 and TRPV4) is linked to profibrotic cardiac effects (Liu et al., 2018; Ahn et al., 2020; Jia et al., 2020), it will be important to determine how the TRPV1 Ca^2+^ signaling could be unique in leading to antifibrosis or if other factors unrelated to Ca^2+^ are involved, so as to identify potential anti-fibrosis drug targets.

In contrast to the protective effects of TRPV1 described above, deleterious effects have also been associated with the presence/activation of these channels. Myocardial hypertrophy with upregulation of TRPV1 expression and loss of cardiac function as well as markers of fibrosis were found to be higher in untreated animals compared to those where TRPV1 was pharmacologically or genetically (TRPV1^−/−^) unfunctional (Buckley and Stokes, 2011). Similarly, Horton et al. (2013) reported that the use of TRPV1 antagonists was associated with reduced fibrosis. The remodeling resulting in scar formation in the infarct border zone was shown to be attenuated by the inactivation of TRPV1 by resiniferatoxin, and was attributed to an effect on adrenergic afferent neurons (Yoshie et al., 2020). The contrasting results in whole animals may be, at least in part, because many cell types, other than cardiac fibroblasts, could be involved in the global effect on cardiac fibrosis observed under pathophysiological or experimental conditions.

#### 3.3.2 TRPV3

The presence of TRPV3 in rat cardiac fibroblasts has been demonstrated through changes in the activity of the channel (Liu et al., 2018). The channel has been implicated in pressure overload-induced cardiac interstitial fibrosis *in vivo* and in the angiotensin II activated, TGF-β1-mediated collagen deposition by fibroblasts *in vitro* (Liu et al., 2018). The *in vivo* and *in vitro* effects could be enhanced by the agonist carvacrol, an effect prevented by the concurrent application of the antagonist ruthenium red, suggesting the implication of TRPV3, but these drugs are known not to be channel specific. The TRPV3 channel activation induces profibrotic effects through Ca^2+^ influx as well as *via* the activation of the fibroblast cell cycle-mediating components called cyclic-dependent kinases and fibroblast proliferation (Liu et al., 2018). Although not many studies have, as yet, addressed the role of TRPV3 in cardiac fibroblasts, the channel is also linked to fibroblast-mediated fibrosis in other tissues such as skin (Um et al., 2020).

#### 3.3.3 TRPV4

The mechanosensitive, Ca^2+^-permeable TRPV4 channel has been characterized in cultured fibroblasts of human (Ahn et al., 2020), rat (Hatano et al., 2009; Adapala et al., 2013; Jia et al., 2020), and porcine (Batan et al., 2022) species, with evidence obtained at the level of channel activity and/or the expression of mRNA or protein. TRPV4 has profibrotic effects in the myocardium through promoting fibroblast-myofibroblast trans-differentiation and fibrinogenesis (Adapala et al., 2013; Ahn et al., 2020; Jia et al., 2020). Post-ischemic fibrotic deposition and associated mechanical dysfunction observed weeks after left anterior descending coronary artery ligation in rats is mediated, at least in part, by TRPV4 since it was less marked in TRPV4 knock-outs, and these effects were mediated *via* TGF-β1 and Rho kinase activation (Adapala et al., 2020). Similarly, osmotically induced increases of intracellular Ca^2+^ in isolated cardiac fibroblasts were also attenuated in cells from TRPV4 silenced models (Adapala et al., 2020). The expression of TRPV4 is also upregulated in the myocardium of diabetic rats (Jia et al., 2020) and in TGF-β1-stimulated human cardiac fibroblasts (Ahn et al., 2020). In turn, the blockade of TRPV4 channel activity down-regulates the expression of TGF-β1 and Smad3 in both the diabetic rat heart and cultured hyperglycaemic fibroblasts (Jia et al., 2020), suggesting that there may be a regulatory cross-talk between TRPV4 and the TGF-β1/Smad3 signaling pathway. Alternatively, the TGF-β1 profibrotic effects linked to TRPV4 in human fibroblast occur *via* the ERK1/2 pathway (Ahn et al., 2020). Furthermore, the activation of TRPV4 channel mediates Ca^2+^ influx in cardiac fibroblasts (Hatano et al., 2009; Ahn et al., 2020) and is linked to the induction of fibroblast-myofibroblast trans-differentiation and diabetic cardiac fibrosis (Ahn et al., 2020; Jia et al., 2020), whereas the inhibition of TRPV4 deactivates myofibroblasts (Batan et al., 2022). TRPV4 also mediates the transformation of other heart cells such as valvular interstitial cells into myofibroblasts *via* the Yes-activated protein (Batan et al., 2022), indicating a broader role of TRPV4 beyond just the functional myocardium to include cardiac valve fibrosis.

### 3.4 TRPA channel

The ankyrin-like channel TRPA1 has long been identified in human lung fibroblasts, where it is implicated in lung fibroblast malignancy (Jaquemar et al., 1999). It has also been shown to be expressed in cardiac fibroblasts of human at mRNA and protein levels (Oguri et al., 2014; Oguri et al., 2021). The channel has also been studied in TRPA1 knockout mice (Li et al., 2019) and is proposed to mediate a TRPA1-like Ca^2+^ current in fibroblasts (Oguri et al., 2021). Furthermore, TRPA1 has been shown to promote cardiac fibroblast proliferation and myofibroblast trans-differentiation (Oguri et al., 2014; Li et al., 2019). These profibrotic effects of TRPA1 are mediated by Ca^2+^ (Oguri et al., 2014; Oguri et al., 2021) as well as through TGF-β1 and the activation of the Ca^2+^-sensitive calcineurin/NFAT signaling pathway (Li et al., 2019).

### 3.5 TRPP channels

TRPP channels assemble either homometrically with themselves or heterometrically with PDK proteins to constitute polycystins. In contrast to the widely recognized role of polycystins in renal structure and function (where polycystin mutations are the cause of polycystic kidney disease, PKD), their role in other tissues, including the myocardium, is less clearly defined. Patients suffering from PKD present with various cardiac abnormalities, indicating that polycystins are also involved in cardiac physiology and pathophysiology. Polycystin 1 (PC1) is present in mouse fibroblasts, where it seems to be localized mainly at the primary cilium detected in these cells, and is implicated in fibroblasts activation (Villalobos et al., 2019). Fibrosis after ischemia is increased under conditions where PC1 is silenced by knockout or RNA-interference, suggesting an antifibrotic action of PC1 (Aranguiz et al., 2021). This protective effect may depend not only on polycystins expressed in fibroblasts but also on those present in cardiomyocytes since a cardiomyocyte-targeted ablation of PC1 is effective to enhance the fibrosis associated with ischemia/reperfusion (Aranguiz et al., 2021). Hence, in addition to the polycystins in fibroblasts mediating ion fluxes, intracellular signal transduction, and autocrine effects on fibroblasts, the polycystins in cardiomyocytes may cause the release of paracrine mediators (e.g., connective tissue growth factor) to modulate fibroblast function.

## 4 TRPs and fibroblast-mediated post-ischemic remodeling

### 4.1 TRPVs and possibly other channels

Given the importance of myocardial ischemia as a major cause of morbidity, especially as related to congestive heart failure, here we summarize the role of fibroblast TRPs in the post-ischemic structural and functional changes (cardiac remodeling).

Cardiac fibroblasts are unique in that they are generally activated by ischemia, whereas the functions of the other permanent myocardial cells such as cardiomyocytes and endothelial cells are depressed by ischemia (Talman and Ruskoaho, 2016). As such, several ischemia-sensitive TRPs in fibroblasts may modulate the subsequent post-ischemic cardiac remodeling. TRPV4 in mouse heart was shown to promote post myocardial infarction-induced ventricular fibrosis that was associated with cardiac dysfunction and poor survival, and those effects were absent in TRPV4 knock-out mice (Adapala et al., 2020). Similarly, the expression of TRPM7, assessed by mRNA and whole-cell currents in single cells, is upregulated post myocardial infarction in mouse cardiac fibroblasts (Li et al., 2008). Furthermore, as a form of cross-talk between fibroblasts and other cardiac tissue components, the presence of TRPV1 in cardiac adrenergic afferent fibres enhanced post myocardial infarction fibrosis in the infarct border zone as well as disrupted connexin connectivity and promoted ventricular arrhythmias (Yoshie et al., 2020). In contrast to profibrotic effects, TRPV1 has also been shown to be cardioprotective against post myocardial infarction, since TRPV1 null-mutant mice had a reduced ejection fraction and a high mortality rate, and were more susceptible to myofibroblast activation and fibrinogenesis (Huang et al., 2010). In addition, TRPC6 has been proposed to be essential for the protective scar formation of the cardiac chamber post myocardial infarction, given that the hearts of TRPC6 null-mutant mice had a smaller protective wall scar and were prone to cardiac rupture and ventricular dilatation as well as to poor functional recovery (Davis et al., 2012)*.* Similarly, TRPP1 was shown to attenuate the fibroblast-myofibroblast differentiation in response to ischemia/reperfusion injury (Aranguiz et al., 2021).

Other TRPs are also probably indirectly linked to myocardial ischemia since ischemia creates a local microenvironment with metabolic disturbances such as hypoxia, acidosis, and oxidative stress, which modulate TRPs in fibroblasts. The evidence for such indirect links is that the profibrotic effects of TRPM7 in cardiac fibroblasts are sensitive to hypoxia (Li et al., 2017), acidosis (Li et al., 2008), and hydrogen peroxide (Guo et al., 2014). Similarly, TRPM2 in cardiac fibroblasts has been shown to be upregulated by hypoxia (Takahashi et al., 2012). Furthermore, the role of TRPs in ischemic remodeling could become broader when considering the possibility of ischemia-induced inflammation. As described above, the profibrotic effects of the pro-inflammatory cytokine TGF-β1 are linked to TRPM7 (Li et al., 2008; Du et al., 2010; Guo et al., 2014; Jia T. et al., 2021) and several other TRPs such as TRPC3, TRPC6, TRPV3, TRPM4 and TRPA1 (Ikeda et al., 2013; Kapur et al., 2014; Liu et al., 2018; He et al., 2019; Li et al., 2019; Ahn et al., 2020; Han et al., 2020; Feng et al., 2021). On the other hand, TRPM7 in cardiac macrophages that are activated during inflammation produces antifibrotic effects (Rios et al., 2020). Therefore, depending on the type of TRP involved, the channels could potentially enhance or attenuate post-ischemic remodeling, but further clarifications are required.

## 5 Fibroblast TRPs and chronic myocardial metal toxicity

Various forms of cardiomyopathies are related to chronic poisoning by metals and include cobalt cardiomyopathy (reported in beer drinkers and in patients with metal hip prostheses) and iron overload cardiomyopathy (known to occur in patients with thalassemia and hemochromatosis), etc. These cardiomyopathies are characterized by metal accumulation into cardiac cells, generally involving cardiomyocytes but it is possible that non-cardiomyocyte cells such as fibroblasts could also be implicated. In general, the entry pathway for the metal is unknown. Although voltage-dependent Ca^2+^ channels have been proposed as pathways for the divalent cation iron (Oudit et al., 2003), pharmacological block of these channels has not suppressed ion entry in some cases, implying that other structures are involved.

Generally, TRP channels mediate the cellular entry of major physiological ions and vital trace elements, but due to their non-selective nature, the channels may also provide an entry pathway for toxic elements. The latter is the case with TRPC6 and TRPM7 channels, which have been shown to mediate cobalt toxicity in rat hearts *in vivo* and in cultured cardiac fibroblasts (Laovitthayanggoon et al., 2019). In that study (Laovitthayanggoon et al., 2019), the cobalt toxicity was associated with an echocardiography-detectable decrease in cardiac contractile function and with an upregulation of the expression of both TRPC6 and TRPM7 in cardiac fibroblasts. However, the cobalt permeability properties of such TRP channels in cardiac fibroblasts have not yet been characterized.

## 6 Challenges

### 6.1 Accuracy of TRP channel identification

A key challenge in inferring the role of TRPs in cardiac fibroblasts has to do with the accuracy of the identification of TRP channels. Although pharmacological probes sufficiently modulate TRPs, the precision of the probes is limited by the general lack of specific blockers and inhibitors of TRP channels. In addition, some TRP channels form hetero-multimers and acquire mixed activation and permeability properties (Cheng et al., 2010) as well as changes in sensitivity to pharmacological probes. For molecular probes, given that some TRPs such as TRPM7 are constitutively active and essential for cell survival (Nadler et al., 2001), TRP knock-out models may induce compensatory pathways of survival that complicate the interpretation of TRP channel effects on fibroblasts. Similarly, the detection of changes in mRNA of TRPs do not necessarily imply altered channel proteins, since the translational processes could unfold differently.

Although some TRPs channels are reported to be present in the heart (mainly in cardiomyocytes), their expression in other non-myocyte cells like fibroblasts may not have been specified. This could be the case with the highly Ca^2+^-selective TRPV5 and TRPV6 channels, which are mainly expressed in epithelial cells, but have also been detected at mRNA and protein levels in the heart (Hwang et al., 2011; Jia X. et al., 2021), and their expression levels have been shown to increase in diabetes experimentally induced by streptozotocin in rat (Jia X. et al., 2021). The cell distribution of TRPV5/6 within the heart, and whether they involve non-cardiomyocyte cells, as well as their function have not, to our knowledge, been investigated. Also, the expression of TRPV2 (which is less investigated in the heart compared to other TRPV channels) was shown to be upregulated in the peri-infarct tissue in response to ischemia, but most markedly in cardiomyocytes and in exogenously infused macrophages (Entin-Meer et al., 2014; Entin-Meer et al., 2017), but direct links to fibroblasts have not been elucidated.

### 6.2 TRP structural complexity

The structural complexity of some TRPs may blur the TRP functionality in fibroblasts. Apart from ion channel activity, some TRPs have metabolically active domains that modulate cardiac fibroblasts, like the kinase in TRPM7 (Rios et al., 2020) and the ADP ribose-binding motif in TRPM2 (Takahashi et al., 2012). These domains may act either in concert with the channel activation or irrespective of the status of channel activation and expression (Takahashi et al., 2012; Rios et al., 2020), thereby making such subtle contributions of the specific TRPs difficult to identify or quantify. Furthermore, other TRPs like TRPV1, TRPV3, and TRPAI have ankyrin repeat domains that are not necessarily metabolically active, but modulate the channel activation (Cordero-Morales et al., 2011; Shi et al., 2013; Ladron-De-Guevara et al., 2020); however, such effects have not yet been studied in cardiac fibroblasts.

### 6.3 Cardiac tissue multicellular interactions

The multicellular myocardial environment in which cardiac fibroblasts reside also limits the understanding of the role of TRPs in fibroblasts. Depending on the species studied and on the type of fibroblast biomarker and quantification methods used, the fraction of fibroblasts relative to the total number of myocardial cells has been reported to be 64% in rats (Banerjee et al., 2007) or 43–58% in humans (Bergmann et al., 2015), or 27% in mice (Banerjee et al., 2007). Such substantial numbers of fibroblasts in the myocardium maximize the possible interactions between fibroblasts and other permanent myocardial cells such as cardiomyocytes, pericytes, and endothelial cells as well as with non-permanent interstitial cells such as macrophages and other immune cells. However, several of these other myocardial cells also express TRP channels (Hof et al., 2019), which may respond to stimuli that are like those that modulate fibroblasts, thereby making it difficult to attribute the contributions of TRP channels specifically to fibroblasts or other myocardial cells. For example, when present in macrophages, the TRPM7 kinase suppresses cardiac fibroblast activity during inflammation (Rios et al., 2020), yet TRPM7 expressed in cardiac fibroblasts has profibrotic effects in other cardiac conditions (Du et al., 2010; Guo et al., 2014; Li et al., 2017; Lu et al., 2017; Wu et al., 2018).

## 7 Summary

Several types of TRPs are expressed in cardiac fibroblasts, in which they induce fibroblast proliferation, fibroblast migration, and myofibroblast differentiation as well as fibrinogenesis in response to not only physiological stimuli, but even more so to pathological stimuli, with the potential to produce cardiac fibrosis, arrhythmias, and post-ischemic pathological cardiac remodeling. Other TRPs have antifibrotic effects, whereas a single type of TRP may have both profibrotic and antifibrotic effects, indicating the complexity of the modulation. Table 1 summarizes the profibrotic and antifibrotic effects of cardiac fibroblast TRPs. Alternatively, cellular influx of non-physiological ions through TRP channels in cardiac fibroblasts may lead to metal toxicity and cardiac contractile dysfunction. Therefore, the modulation of cardiac fibroblasts by TRPs contributes to baseline fibroblastic activity, beneficial myocardial healing, and to cardiac disease processes.

**TABLE 1:** Profibrotic and antifibrotic effects of TRP channels in cardiac fibroblasts.

TRP	Profibrosis (fibroblast species, references)	Antifibrosis (fibroblast species, references)
TRPC3	Human (Harada et al., 2012; Saliba et al., 2019; Han et al., 2020)	—
	Rat (Numaga-Tomita et al., 2016; He et al., 2019; Saliba et al., 2019)	
	Mouse (Numaga-Tomita et al., 2016; Han et al., 2020)	
	Canine (Harada et al., 2012)	
TRPC6	Human (Ikeda et al., 2013; Kapur et al., 2014)	Rat (Nishida et al., 2007)
	Mouse (Davis et al., 2012; Kapur et al., 2014)	
TRPM2	Rat (Takahashi et al., 2012)	—
TRPM4	Human (Feng et al., 2021)	—
TRPM7	Human (Du et al., 2010)	^a^Mouse (Rios et al., 2020)
	Rat (Yu Y. et al., 2014; Guo et al., 2014; Zhou et al., 2015; Li et al., 2017; Lu et al., 2017; Zhong H. et al., 2018b; Wu et al., 2018; Jia T. et al., 2021)	
	Mouse (Li et al., 2008; Jia X. et al., 2021)	
TRPV1	Mouse (Buckley and Stokes, 2011; Horton et al., 2013)	Mouse (Huang et al., 2010; Wang et al., 2014; Wang et al., 2016; Zhong B. et al., 2018)
	^b^Porcine (Yoshie et al., 2020)	
TRPV3	Rat (Liu et al., 2018)	—
TRPV4	Human (Ahn et al., 2020)	—
	Rat (Hatano et al., 2009; Adapala et al., 2013; Jia et al., 2020)	
	Porcine (Batan et al., 2022)	
TRPA1	Human (Oguri et al., 2014; Oguri et al., 2021)	—
	Mouse (Li et al., 2019)	
TRPP1	Mouse (Villalobos et al., 2019)	Mouse (Aranguiz et al., 2021)
	—	Rat (Aranguiz et al., 2021)

a TRP, in cardiac macrophages.

b TRP, in cardiac sympathetic afferents.

**FIGURE 1:**
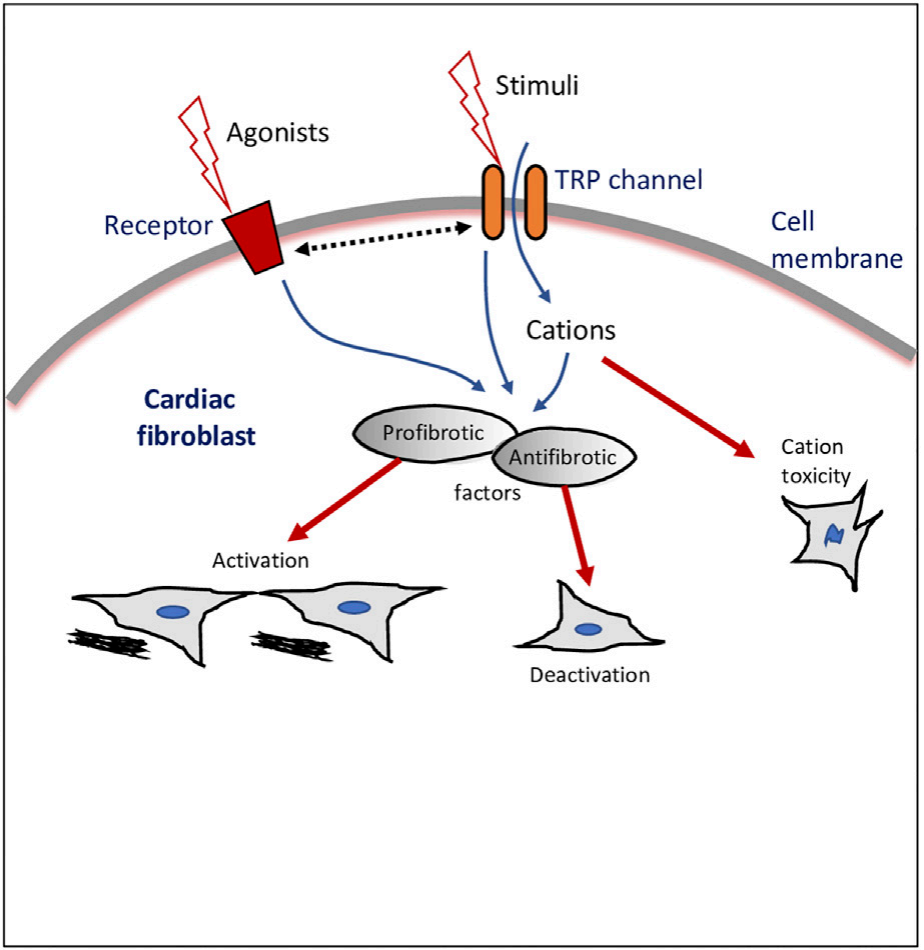
Transient receptor potential (TRP) ion channel modulation of cardiac fibroblasts. Schematic drawing of a cardiac fibroblast, external stimulants, and intracellular cascades and outcomes of TRP channels-mediated effects. Dotted line with double arrowheads depicts crosstalk or bi-directional modulation.

The TRPs in fibroblasts act as sensors of various stimuli and as pathways of entry of Ca^2+^ and other cations as well as modulate either profibrotic or antifibrotic mediators (Figure 1). Nonetheless, the molecular mechanisms involved in TRP-mediated activation or de-activation of fibroblasts/myofibroblasts, and the role of TRPs in cell-cell interactions of fibroblasts with other cardiac cells still require further studies. Therefore, the recognition of the existence of TRPs in fibroblasts is an emerging area of cardiac pathophysiology, with the potential to unlock the broad functionality of fibroblasts and the identification of novel therapeutic drug targets in cardiac disease.

## References

[B1] Adapala R. K. Kanugula A. K. Paruchuri S. Chilian W. M. Thodeti C. K. (2020). TRPV4 deletion protects heart from myocardial infarction-induced adverse remodeling via modulation of cardiac fibroblast differentiation. Basic Res. Cardiol. 115, 14. 10.1007/s00395-020-0775-5 31925567 PMC7322630

[B2] Adapala R. K. Thoppil R. J. Luther D. J. Paruchuri S. Meszaros J. G. Chilian W. M. (2013). TRPV4 channels mediate cardiac fibroblast differentiation by integrating mechanical and soluble signals. J. Mol. Cell. Cardiol. 54, 45–52. 10.1016/j.yjmcc.2012.10.016 23142541 PMC3935769

[B3] Ahn M. S. Eom Y. W. Oh J. E. Cha S. K. Park K. S. Son J. W. (2020). Transient receptor potential channel TRPV4 mediates TGF-β1-induced differentiation of human ventricular fibroblasts. Cardiol. J. 27, 162–170. 10.5603/CJ.a2019.0050 32329036 PMC8016025

[B4] Alatrag F. Amoni M. Kelly-Laubscher R. Gwanyanya A. (2022). Cardioprotective effect of fingolimod against calcium paradox-induced myocardial injury in the isolated rat heart. Can. J. Physiol. Pharmacol. 100, 134–141. 10.1139/cjpp-2021-0381 34559972

[B5] Albert A. P. (2011). Gating mechanisms of canonical transient receptor potential channel proteins: Role of phosphoinositols and diacylglycerol. Adv. Exp. Med. Biol. 704, 391–411. 10.1007/978-94-007-0265-3_22 21290308

[B6] Andriule I. Pangonyte D. Almanaityte M. Patamsyte V. Kupryte M. Karciauskas D. (2021). Evidence for the expression of TRPM6 and TRPM7 in cardiomyocytes from all four chamber walls of the human heart. Sci. Rep. 11, 15445. 10.1038/s41598-021-94856-4 34326388 PMC8322396

[B7] Aranguiz P. Romero P. Vasquez F. Flores-Vergara R. Aravena D. Sanchez G. (2021). Polycystin-1 mitigates damage and regulates CTGF expression through AKT activation during cardiac ischemia/reperfusion. Biochim. Biophys. Acta. Mol. Basis Dis. 1867, 165986. 10.1016/j.bbadis.2020.165986 33065236

[B8] Aujla P. K. Kassiri Z. (2021). Diverse origins and activation of fibroblasts in cardiac fibrosis. Cell. Signal. 78, 109869. 10.1016/j.cellsig.2020.109869 33278559

[B9] Banerjee I. Fuseler J. W. Price R. L. Borg T. K. Baudino T. A. (2007). Determination of cell types and numbers during cardiac development in the neonatal and adult rat and mouse. Am. J. Physiol. Heart Circ. Physiol. 293, H1883–H1891. 10.1152/ajpheart.00514.2007 17604329

[B10] Batan D. Peters D. K. Schroeder M. E. Aguado B. A. Young M. W. Weiss R. M. (2022). Hydrogel cultures reveal transient receptor potential vanilloid 4 regulation of myofibroblast activation and proliferation in valvular interstitial cells. FASEB J. 36, e22306. 10.1096/fj.202101863R 35385164 PMC9009405

[B11] Bergmann O. Zdunek S. Felker A. Salehpour M. Alkass K. Bernard S. (2015). Dynamics of cell generation and turnover in the human heart. Cell 161, 1566–1575. 10.1016/j.cell.2015.05.026 26073943

[B12] Buckley C. L. Stokes A. J. (2011). Mice lacking functional TRPV1 are protected from pressure overload cardiac hypertrophy. Channels (Austin) 5, 367–374. 10.4161/chan.5.4.17083 21814047 PMC3225734

[B13] Camacho Londono J. E. Marx A. Kraft A. E. Schurger A. Richter C. Dietrich A. (2020). Angiotensin-II-evoked Ca^2+^ entry in murine cardiac fibroblasts does not depend on TRPC channels. Cells 9, E322. 10.3390/cells9020322 32013125 PMC7072683

[B14] Camacho Londono J. E. Tian Q. Hammer K. Schroder L. Camacho Londono J. Reil J. C. (2015). A background Ca^2+^ entry pathway mediated by TRPC1/TRPC4 is critical for development of pathological cardiac remodelling. Eur. Heart J. 36, 2257–2266. 10.1093/eurheartj/ehv250 26069213 PMC4554959

[B15] Cappetta D. Esposito G. Piegari E. Russo R. Ciuffreda L. P. Rivellino A. (2016). SIRT1 activation attenuates diastolic dysfunction by reducing cardiac fibrosis in a model of anthracycline cardiomyopathy. Int. J. Cardiol. 205, 99–110. 10.1016/j.ijcard.2015.12.008 26730840

[B16] Certal M. Vinhas A. Barros-Barbosa A. Ferreirinha F. Costa M. A. Correia-De-Sa P. (2017). ADP-induced Ca^2+^ signaling and proliferation of rat ventricular myofibroblasts depend on phospholipase C-Linked TRP channels activation within lipid rafts. J. Cell. Physiol. 232, 1511–1526. 10.1002/jcp.25656 27755650

[B17] Chan E. C. Peshavariya H. M. Liu G. S. Jiang F. Lim S. Y. Dusting G. J. (2013). Nox4 modulates collagen production stimulated by transforming growth factor β1 *in vivo* and *in vitro*. Biochem. Biophys. Res. Commun. 430, 918–925. 10.1016/j.bbrc.2012.11.138 23261430

[B18] Chandy M. (2019). A tangled tale of microRNA and cardiac fibrosis. Clin. Sci. 133, 2217–2220. 10.1042/CS20190866 31722012

[B19] Chen Q. Q. Zhang W. Chen X. F. Bao Y. J. Wang J. Zhu W. Z. (2012). Electrical field stimulation induces cardiac fibroblast proliferation through the calcineurin-NFAT pathway. Can. J. Physiol. Pharmacol. 90, 1611–1622. 10.1139/y2012-133 23210440

[B20] Cheng K. T. Ong H. L. Liu X. Ambudkar I. S. (2013). Contribution and regulation of TRPC channels in store-operated Ca^2+^ entry. Curr. Top. Membr. 71, 149–179. 10.1016/B978-0-12-407870-3.00007-X 23890115 PMC3824975

[B21] Cheng W. Sun C. Zheng J. (2010). Heteromerization of TRP channel subunits: Extending functional diversity. Protein Cell 1, 802–810. 10.1007/s13238-010-0108-9 21203922 PMC4875230

[B22] Chung C. C. Lin Y. K. Chen Y. C. Kao Y. H. Yeh Y. H. Chen Y. J. (2021). Calcium regulation on the atrial regional difference of collagen production activity in atrial fibrogenesis. Biomedicines 9, 686. 10.3390/biomedicines9060686 34204537 PMC8233809

[B23] Cordero-Morales J. F. Gracheva E. O. Julius D. (2011). Cytoplasmic ankyrin repeats of transient receptor potential A1 (TRPA1) dictate sensitivity to thermal and chemical stimuli. Proc. Natl. Acad. Sci. U. S. A. 108, E1184–E1191. 10.1073/pnas.1114124108 21930928 PMC3219143

[B24] Davis J. Burr A. R. Davis G. F. Birnbaumer L. Molkentin J. D. (2012). A TRPC6-dependent pathway for myofibroblast transdifferentiation and wound healing *in vivo* . Dev. Cell 23, 705–715. 10.1016/j.devcel.2012.08.017 23022034 PMC3505601

[B25] Davis J. Molkentin J. D. (2014). Myofibroblasts: Trust your heart and let fate decide. J. Mol. Cell. Cardiol. 70, 9–18. 10.1016/j.yjmcc.2013.10.019 24189039 PMC3995855

[B26] Deleon-Pennell K. Y. Barker T. H. Lindsey M. L. (2020). Fibroblasts: The arbiters of extracellular matrix remodeling. Matrix Biol. 91-92, 1–7. 10.1016/j.matbio.2020.05.006 32504772 PMC7434687

[B27] Du J. Xie J. Zhang Z. Tsujikawa H. Fusco D. Silverman D. (2010). TRPM7-mediated Ca^2+^ signals confer fibrogenesis in human atrial fibrillation. Circ. Res. 106, 992–1003. 10.1161/CIRCRESAHA.109.206771 20075334 PMC2907241

[B28] Du L. Qin M. Yi Y. Chen X. Jiang W. Zhou L. (2017). Eplerenone prevents atrial fibrosis via the TGF-beta signaling pathway. Cardiology 138, 55–62. 10.1159/000471918 28571007

[B29] Dzialo E. Czepiel M. Tkacz K. Siedlar M. Kania G. Blyszczuk P. (2021). WNT/β-Catenin signaling promotes TGF-β-mediated activation of human cardiac fibroblasts by enhancing IL-11 production. Int. J. Mol. Sci. 22, 10072. 10.3390/ijms221810072 34576234 PMC8468519

[B30] Entin-Meer M. Cohen L. Hertzberg-Bigelman E. Levy R. Ben-Shoshan J. Keren G. (2017). TRPV2 knockout mice demonstrate an improved cardiac performance following myocardial infarction due to attenuated activity of peri-infarct macrophages. PLoS One 12, e0177132. 10.1371/journal.pone.0177132 28481959 PMC5421795

[B31] Entin-Meer M. Levy R. Goryainov P. Landa N. Barshack I. Avivi C. (2014). The transient receptor potential vanilloid 2 cation channel is abundant in macrophages accumulating at the peri-infarct zone and may enhance their migration capacity towards injured cardiomyocytes following myocardial infarction. PLoS One 9, e105055. 10.1371/journal.pone.0105055 25136832 PMC4138115

[B32] Feng J. Zong P. Yan J. Yue Z. Li X. Smith C. (2021). Upregulation of transient receptor potential melastatin 4 (TRPM4) in ventricular fibroblasts from heart failure patients. Pflugers Arch. 473, 521–531. 10.1007/s00424-021-02525-2 33594499 PMC8857941

[B33] Feng Y. Bao Y. Ding J. Li H. Liu W. Wang X. (2022). MicroRNA-130a attenuates cardiac fibrosis after myocardial infarction through TGF-β/Smad signaling by directly targeting TGF-β receptor 1. Bioengineered 13, 5779–5791. 10.1080/21655979.2022.2033380 35188441 PMC8973730

[B34] Fix C. Bingham K. Carver W. (2011). Effects of interleukin-18 on cardiac fibroblast function and gene expression. Cytokine 53, 19–28. 10.1016/j.cyto.2010.10.002 21050772 PMC3018826

[B35] Frangogiannis N. G. (2019). Cardiac fibrosis: Cell biological mechanisms, molecular pathways and therapeutic opportunities. Mol. Asp. Med. 65, 70–99. 10.1016/j.mam.2018.07.001 30056242

[B36] Freichel M. Berlin M. Schurger A. Mathar I. Bacmeister L. Medert R. (2017). “TRP channels in the heart,” in Neurobiology of TRP channels. Editor Emir T. L. R., 149–185. 10.4324/9781315152837-9 29356479

[B37] Gao H. Bo Z. Wang Q. Luo L. Zhu H. Ren Y. (2019). Salvanic acid B inhibits myocardial fibrosis through regulating TGF-β1/Smad signaling pathway. Biomed. Pharmacother. 110, 685–691. 10.1016/j.biopha.2018.11.098 30553195

[B38] Gaudesius G. Miragoli M. Thomas S. P. Rohr S. (2003). Coupling of cardiac electrical activity over extended distances by fibroblasts of cardiac origin. Circ. Res. 93, 421–428. 10.1161/01.RES.0000089258.40661.0C 12893743

[B39] Gaudet R. (2007). “''Structural insights into the function of TRP channels,” in TRP ion channel function in sensory transduction and cellular signaling cascades. Editors Liedtke W. B. Heller S.. Chapter 25.

[B40] Gevaert A. B. Boen J. R. A. Segers V. F. Van Craenenbroeck E. M. (2019). Heart failure with preserved ejection fraction: A review of cardiac and noncardiac pathophysiology. Front. Physiol. 10, 638. 10.3389/fphys.2019.00638 31191343 PMC6548802

[B41] Guinamard R. Demion M. Magaud C. Potreau D. Bois P. (2006). Functional expression of the TRPM4 cationic current in ventricular cardiomyocytes from spontaneously hypertensive rats. Hypertension 48, 587–594. 10.1161/01.HYP.0000237864.65019.a5 16966582

[B42] Guinamard R. Salle L. Simard C. (2011). The non-selective monovalent cationic channels TRPM4 and TRPM5. Adv. Exp. Med. Biol. 704, 147–171. 10.1007/978-94-007-0265-3_8 21290294

[B43] Guo J. L. Yu Y. Jia Y. Y. Ma Y. Z. Zhang B. Y. Liu P. Q. (2014). Transient receptor potential melastatin 7 (TRPM7) contributes to H_2_O_2_-induced cardiac fibrosis via mediating Ca^2+^ influx and extracellular signal-regulated kinase 1/2 (ERK1/2) activation in cardiac fibroblasts. J. Pharmacol. Sci. 125, 184–192. 10.1254/jphs.13224fp 24871786

[B44] Gwanyanya A. Andriule I. Istrate B. M. Easmin F. Mubagwa K. Macianskiene R. (2021). Modulation of the cardiac myocyte action potential by the magnesium-sensitive TRPM6 and TRPM7-like current. Int. J. Mol. Sci. 22, 8744. 10.3390/ijms22168744 34445449 PMC8395930

[B45] Hall C. Gehmlich K. Denning C. Pavlovic D. (2021). Complex relationship between cardiac fibroblasts and cardiomyocytes in health and disease. J. Am. Heart Assoc. 10, e019338. 10.1161/JAHA.120.019338 33586463 PMC8174279

[B46] Han L. Tang Y. Li S. Wu Y. Chen X. Wu Q. (2020). Protective mechanism of SIRT1 on Hcy-induced atrial fibrosis mediated by TRPC3. J. Cell. Mol. Med. 24, 488–510. 10.1111/jcmm.14757 31680473 PMC6933351

[B47] Hanna A. Humeres C. Frangogiannis N. G. (2021). The role of Smad signaling cascades in cardiac fibrosis. Cell. Signal. 77, 109826. 10.1016/j.cellsig.2020.109826 33160018 PMC7727442

[B48] Harada M. Luo X. Qi X. Y. Tadevosyan A. Maguy A. Ordog B. (2012). Transient receptor potential canonical-3 channel-dependent fibroblast regulation in atrial fibrillation. Circulation 126, 2051–2064. 10.1161/CIRCULATIONAHA.112.121830 22992321 PMC3675169

[B49] Hardie R. C. Minke B. (1992). The trp gene is essential for a light-activated Ca^2+^ channel in Drosophila photoreceptors. Neuron 8, 643–651. 10.1016/0896-6273(92)90086-s 1314617

[B50] Hatano N. Itoh Y. Muraki K. (2009). Cardiac fibroblasts have functional TRPV4 activated by 4alpha-phorbol 12, 13-didecanoate. Life Sci. 85, 808–814. 10.1016/j.lfs.2009.10.013 19879881

[B51] He R. Zhang J. Luo D. Yu Y. Chen T. Yang Y. (2019). Upregulation of transient receptor potential canonical type 3 channel via at1r/TGF-β1/smad2/3 induces atrial fibrosis in aging and spontaneously hypertensive rats. Oxid. Med. Cell. Longev. 2019, 4025496. 10.1155/2019/4025496 31871548 PMC6906806

[B52] Hof T. Chaigne S. Recalde A. Salle L. Brette F. Guinamard R. (2019). Transient receptor potential channels in cardiac health and disease. Nat. Rev. Cardiol. 16, 344–360. 10.1038/s41569-018-0145-2 30664669

[B53] Hofmann K. Fiedler S. Vierkotten S. Weber J. Klee S. Jia J. (2017). Classical transient receptor potential 6 (TRPC6) channels support myofibroblast differentiation and development of experimental pulmonary fibrosis. Biochim. Biophys. Acta. Mol. Basis Dis. 1863, 560–568. 10.1016/j.bbadis.2016.12.002 27932059

[B54] Horton J. S. Buckley C. L. Stokes A. J. (2013). Successful TRPV1 antagonist treatment for cardiac hypertrophy and heart failure in mice. Channels (Austin) 7, 17–22. 10.4161/chan.23006 23221478 PMC3589277

[B55] Huang H. Wang W. Liu P. Jiang Y. Zhao Y. Wei H. (2009). TRPC1 expression and distribution in rat hearts. Eur. J. Histochem. 53, e26. 10.4081/ejh.2009.e26 22073358 PMC3167335

[B56] Huang J. Zhang X. Mcnaughton P. A. (2006). Modulation of temperature-sensitive TRP channels. Semin. Cell Dev. Biol. 17, 638–645. 10.1016/j.semcdb.2006.11.002 17185012

[B57] Huang W. Rubinstein J. Prieto A. R. Wang D. H. (2010). Enhanced postmyocardial infarction fibrosis via stimulation of the transforming growth factor-beta-Smad2 signaling pathway: Role of transient receptor potential vanilloid type 1 channels. J. Hypertens. 28, 367–376. 10.1097/HJH.0b013e328333af48 19887954

[B58] Hwang I. Jung E. M. Yang H. Choi K. C. Jeung E. B. (2011). Tissue-specific expression of the calcium transporter genes TRPV5, TRPV6, NCX1, and PMCA1b in the duodenum, kidney and heart of *Equus caballus* . J. Vet. Med. Sci. 73, 1437–1444. 10.1292/jvms.11-0141 21737966

[B59] Ikeda K. Nakajima T. Yamamoto Y. Takano N. Tanaka T. Kikuchi H. (2013). Roles of transient receptor potential canonical (TRPC) channels and reverse-mode Na^+^/Ca^2+^ exchanger on cell proliferation in human cardiac fibroblasts: Effects of transforming growth factor beta1. Cell Calcium 54, 213–225. 10.1016/j.ceca.2013.06.005 23827314

[B60] Inoue R. Jian Z. Kawarabayashi Y. (2009). Mechanosensitive TRP channels in cardiovascular pathophysiology. Pharmacol. Ther. 123, 371–385. 10.1016/j.pharmthera.2009.05.009 19501617

[B61] Inoue R. Kurahara L. H. Hiraishi K. (2019). TRP channels in cardiac and intestinal fibrosis. Semin. Cell Dev. Biol. 94, 40–49. 10.1016/j.semcdb.2018.11.002 30445149

[B62] Jaquemar D. Schenker T. Trueb B. (1999). An ankyrin-like protein with transmembrane domains is specifically lost after oncogenic transformation of human fibroblasts. J. Biol. Chem. 274, 7325–7333. 10.1074/jbc.274.11.7325 10066796

[B63] Jatho A. Hartmann S. Kittana N. Mugge F. Wuertz C. M. Tiburcy M. (2015). RhoA ambivalently controls prominent myofibroblast characteritics by involving distinct signaling routes. PLoS One 10, e0137519. 10.1371/journal.pone.0137519 26448568 PMC4598021

[B64] Jia T. Wang X. Tang Y. Yu W. Li C. Cui S. (2021). Sacubitril ameliorates cardiac fibrosis through inhibiting TRPM7 channel. Front. Cell Dev. Biol. 9, 760035. 10.3389/fcell.2021.760035 34778271 PMC8586221

[B65] Jia X. Xiao C. Sheng D. Yang M. Cheng Q. Wu J. (2020). TRPV4 mediates cardiac fibrosis via the TGF-β1/smad3 signaling pathway in diabetic rats. Cardiovasc. Toxicol. 20, 492–499. 10.1007/s12012-020-09572-8 32274619

[B66] Jia X. X. Yu T. Xiao C. Sheng D. Yang M. Cheng Q. (2021). Expression of transient receptor potential vanilloid genes and proteins in diabetic rat heart. Mol. Biol. Rep. 48, 1217–1223. 10.1007/s11033-021-06182-7 33523372

[B67] Kamimura D. Ohtani T. Sakata Y. Mano T. Takeda Y. Tamaki S. (2012). Ca^2+^ entry mode of Na^+^/Ca^2+^ exchanger as a new therapeutic target for heart failure with preserved ejection fraction. Eur. Heart J. 33, 1408–1416. 10.1093/eurheartj/ehr106 21490055

[B68] Kapoun A. M. Liang F. O'young G. Damm D. L. Quon D. White R. T. (2004). B-Type natriuretic peptide exerts broad functional opposition to transforming growth factor-beta in primary human cardiac fibroblasts: Fibrosis, myofibroblast conversion, proliferation, and inflammation. Circ. Res. 94, 453–461. 10.1161/01.RES.0000117070.86556.9F 14726474

[B69] Kapur N. K. Qiao X. Paruchuri V. Mackey E. E. Daly G. H. Ughreja K. (2014). Reducing endoglin activity limits calcineurin and TRPC-6 expression and improves survival in a mouse model of right ventricular pressure overload. J. Am. Heart Assoc. 3, e000965. 10.1161/JAHA.114.000965 25015075 PMC4310384

[B70] Katwa L. C. (2003). Cardiac myofibroblasts isolated from the site of myocardial infarction express endothelin de novo. Am. J. Physiol. Heart Circ. Physiol. 285, H1132–H1139. 10.1152/ajpheart.01141.2002 12738614 PMC3892894

[B71] Kohl P. Camelliti P. Burton F. L. Smith G. L. (2005). Electrical coupling of fibroblasts and myocytes: Relevance for cardiac propagation. J. Electrocardiol. 38, 45–50. 10.1016/j.jelectrocard.2005.06.096 16226073

[B72] Ladron-De-Guevara E. Dominguez L. Rangel-Yescas G. E. Fernandez-Velasco D. A. Torres-Larios A. Rosenbaum T. (2020). The contribution of the ankyrin repeat domain of TRPV1 as a thermal module. Biophys. J. 118, 836–845. 10.1016/j.bpj.2019.10.041 31757360 PMC7036727

[B73] Laovitthayanggoon S. Henderson C. J. Mccluskey C. Macdonald M. Tate R. J. Grant M. H. (2019). Cobalt administration causes reduced contractility with parallel increases in TRPC6 and TRPM7 transporter protein expression in adult rat hearts. Cardiovasc. Toxicol. 19, 276–286. 10.1007/s12012-018-9498-3 30523498 PMC6505488

[B74] Levick S. P. Murray D. B. Janicki J. S. Brower G. L. (2010). Sympathetic nervous system modulation of inflammation and remodeling in the hypertensive heart. Hypertension 55, 270–276. 10.1161/HYPERTENSIONAHA.109.142042 20048196 PMC2823485

[B75] Li M. J. Zhou Y. M. Tang Y. H. Cao F. (2008). Increased expression of transient receptor potential melastatin 7 in mouse cardiac fibroblasts post myocardial infarction. Zhonghua Xin Xue Guan Bing Za Zhi 36, 641–645. 19100096

[B76] Li S. Li M. Yi X. Guo F. Zhou Y. Chen S. (2017). TRPM7 channels mediate the functional changes in cardiac fibroblasts induced by angiotensin II. Int. J. Mol. Med. 39, 1291–1298. 10.3892/ijmm.2017.2943 28393175

[B77] Li S. Sun X. Wu H. Yu P. Wang X. Jiang Z. (2019). TRPA1 promotes cardiac myofibroblast transdifferentiation after myocardial infarction injury via the calcineurin-NFAT-DYRK1A signaling pathway. Oxid. Med. Cell. Longev. 2019, 6408352. 10.1155/2019/6408352 31217840 PMC6537015

[B78] Liu C. Montell C. (2015). Forcing open TRP channels: Mechanical gating as a unifying activation mechanism. Biochem. Biophys. Res. Commun. 460, 22–25. 10.1016/j.bbrc.2015.02.067 25998730 PMC4441759

[B79] Liu H. El Zein L. Kruse M. Guinamard R. Beckmann A. Bozio A. (2010). Gain-of-function mutations in TRPM4 cause autosomal dominant isolated cardiac conduction disease. Circ. Cardiovasc. Genet. 3, 374–385. 10.1161/CIRCGENETICS.109.930867 20562447

[B80] Liu Q. Chen Y. Auger-Messier M. Molkentin J. D. (2012). Interaction between NFκB and NFAT coordinates cardiac hypertrophy and pathological remodeling. Circ. Res. 110, 1077–1086. 10.1161/CIRCRESAHA.111.260729 22403241 PMC3341669

[B81] Liu Y. Qi H. E M. Shi P. Zhang Q. Li S. (2018). Transient receptor potential vanilloid-3 (TRPV3) activation plays a central role in cardiac fibrosis induced by pressure overload in rats via TGF-β1 pathway. Schmiedeb. Arch. Pharmacol. 391, 131–143. 10.1007/s00210-017-1443-7 29249037

[B82] Lu J. Wang Q. Y. Zhou Y. Lu X. C. Liu Y. H. Wu Y. (2017). Astragaloside against cardiac fibrosis by inhibiting TRPM7 channel. Phytomedicine 30, 10–17. 10.1016/j.phymed.2017.04.002 28545665

[B83] Macianskiene R. Almanaityte M. Jekabsone A. Mubagwa K. (2017). Modulation of human cardiac TRPM7 current by extracellular acidic pH depends upon extracellular concentrations of divalent cations. PLoS One 12, e0170923. 10.1371/journal.pone.0170923 28129376 PMC5271359

[B84] Maity S. Muhamed J. Sarikhani M. Kumar S. Ahamed F. Spurthi K. M. (2020). Sirtuin 6 deficiency transcriptionally up-regulates TGF-beta signaling and induces fibrosis in mice. J. Biol. Chem. 295, 415–434. 10.1074/jbc.RA118.007212 31744885 PMC6956532

[B85] Miragoli M. Gaudesius G. Rohr S. (2006). Electrotonic modulation of cardiac impulse conduction by myofibroblasts. Circ. Res. 98, 801–810. 10.1161/01.RES.0000214537.44195.a3 16484613

[B86] Moore-Morris T. Guimaraes-Camboa N. Yutzey K. E. Puceat M. Evans S. M. (2015). Cardiac fibroblasts: From development to heart failure. J. Mol. Med. 93, 823–830. 10.1007/s00109-015-1314-y 26169532 PMC4512919

[B87] Nadler M. J. Hermosura M. C. Inabe K. Perraud A. L. Zhu Q. Stokes A. J. (2001). LTRPC7 is a Mg.ATP-regulated divalent cation channel required for cell viability. Nature 411, 590–595. 10.1038/35079092 11385574

[B88] Nikolaev Y. A. Cox C. D. Ridone P. Rohde P. R. Cordero-Morales J. F. Vasquez V. (2019). Mammalian TRP ion channels are insensitive to membrane stretch. J. Cell Sci. 132, jcs238360. 10.1242/jcs.238360 31722978 PMC6918743

[B89] Nilius B. Owsianik G. (2011). The transient receptor potential family of ion channels. Genome Biol. 12, 218. 10.1186/gb-2011-12-3-218 21401968 PMC3129667

[B90] Nishida M. Onohara N. Sato Y. Suda R. Ogushi M. Tanabe S. (2007). Galpha12/13-mediated up-regulation of TRPC6 negatively regulates endothelin-1-induced cardiac myofibroblast formation and collagen synthesis through nuclear factor of activated T cells activation. J. Biol. Chem. 282, 23117–23128. 10.1074/jbc.M611780200 17533154

[B91] Numaga-Tomita T. Kitajima N. Kuroda T. Nishimura A. Miyano K. Yasuda S. (2016). TRPC3-GEF-H1 axis mediates pressure overload-induced cardiac fibrosis. Sci. Rep. 6, 39383. 10.1038/srep39383 27991560 PMC5171702

[B92] Numaga-Tomita T. Oda S. Shimauchi T. Nishimura A. Mangmool S. Nishida M. (2017). TRPC3 channels in cardiac fibrosis. Front. Cardiovasc. Med. 4, 56. 10.3389/fcvm.2017.00056 28936433 PMC5594069

[B93] Oguri G. Nakajima T. Kikuchi H. Obi S. Nakamura F. Komuro I. (2021). Allyl isothiocyanate (AITC) activates nonselective cation currents in human cardiac fibroblasts: Possible involvement of TRPA1. Heliyon 7, e05816. 10.1016/j.heliyon.2020.e05816 33458442 PMC7797518

[B94] Oguri G. Nakajima T. Yamamoto Y. Takano N. Tanaka T. Kikuchi H. (2014). Effects of methylglyoxal on human cardiac fibroblast: Roles of transient receptor potential ankyrin 1 (TRPA1) channels. Am. J. Physiol. Heart Circ. Physiol. 307, H1339–H1352. 10.1152/ajpheart.01021.2013 25172898

[B95] Ongstad E. Kohl P. (2016). Fibroblast-myocyte coupling in the heart: Potential relevance for therapeutic interventions. J. Mol. Cell. Cardiol. 91, 238–246. 10.1016/j.yjmcc.2016.01.010 26774702 PMC5022561

[B96] Oudit G. Y. Sun H. Trivieri M. G. Koch S. E. Dawood F. Ackerley C. (2003). L-type Ca^2+^ channels provide a major pathway for iron entry into cardiomyocytes in iron-overload cardiomyopathy. Nat. Med. 9, 1187–1194. 10.1038/nm920 12937413

[B97] Peng J. B. Suzuki Y. Gyimesi G. Hediger M. A. (2018). “''TRPV5 and TRPV6 calcium-selective channels,” in Calcium entry channels in non-excitable cells. Editors Kozak J. A. Jr. J. W. (Putney, 241–274. 10.1201/9781315152592-13 30299660

[B98] Petigny C. Dumont A. A. Giguere H. Collette A. Holleran B. J. Iftinca M. (2022). Monitoring TRPC7 conformational changes by BRET following GPCR activation. Int. J. Mol. Sci. 23, 2502. 10.3390/ijms23052502 35269644 PMC8910688

[B99] Revell C. M. Dietrich J. A. Scott C. C. Luttge A. Baggett L. S. Athanasiou K. A. (2006). Characterization of fibroblast morphology on bioactive surfaces using vertical scanning interferometry. Matrix Biol. 25, 523–533. 10.1016/j.matbio.2006.07.007 16962756

[B100] Rios F. J. Zou Z. G. Harvey A. P. Harvey K. Y. Nosalski R. Anyfanti P. (2020). Chanzyme TRPM7 protects against cardiovascular inflammation and fibrosis. Cardiovasc. Res. 116, 721–735. 10.1093/cvr/cvz164 31250885 PMC7252442

[B101] Rosenkranz S. (2004). TGF-beta1 and angiotensin networking in cardiac remodeling. Cardiovasc. Res. 63, 423–432. 10.1016/j.cardiores.2004.04.030 15276467

[B102] Runnels L. W. Yue L. Clapham D. E. (2001). TRP-PLIK, a bifunctional protein with kinase and ion channel activities. Science 291, 1043–1047. 10.1126/science.1058519 11161216

[B103] Saliba Y. Jebara V. Hajal J. Maroun R. Chacar S. Smayra V. (2019). Transient receptor potential canonical 3 and nuclear factor of activated T cells C3 signaling pathway critically regulates myocardial fibrosis. Antioxid. Redox Signal. 30, 1851–1879. 10.1089/ars.2018.7545 30318928 PMC6486676

[B104] Satoh H. Delbridge L. M. Blatter L. A. Bers D. M. (1996). Surface:volume relationship in cardiac myocytes studied with confocal microscopy and membrane capacitance measurements: Species-dependence and developmental effects. Biophys. J. 70, 1494–1504. 10.1016/S0006-3495(96)79711-4 8785306 PMC1225076

[B105] Schafer S. Viswanathan S. Widjaja A. A. Lim W. W. Moreno-Moral A. Delaughter D. M. (2017). IL-11 is a crucial determinant of cardiovascular fibrosis. Nature 552, 110–115. 10.1038/nature24676 29160304 PMC5807082

[B106] Schimmel K. Ichimura K. Reddy S. Haddad F. Spiekerkoetter E. (2022). Cardiac fibrosis in the pressure overloaded left and right ventricle as a therapeutic target. Front. Cardiovasc. Med. 9, 886553. 10.3389/fcvm.2022.886553 35600469 PMC9120363

[B107] Shi D. J. Ye S. Cao X. Zhang R. Wang K. (2013). Crystal structure of the N-terminal ankyrin repeat domain of TRPV3 reveals unique conformation of finger 3 loop critical for channel function. Protein Cell 4, 942–950. 10.1007/s13238-013-3091-0 24248473 PMC4875401

[B108] Soliman H. Rossi F. M. V. (2020). Cardiac fibroblast diversity in health and disease. Matrix Biol. 91-92, 75–91. 10.1016/j.matbio.2020.05.003 32446910

[B109] Squier C. A. Ghoneim S. Kremenak C. R. (1990). Ultrastructure of the periosteum from membrane bone. J. Anat. 171, 233–239. 2081707 PMC1257144

[B110] Sridhar S. Vandersickel N. Panfilov A. V. (2017). Effect of myocyte-fibroblast coupling on the onset of pathological dynamics in a model of ventricular tissue. Sci. Rep. 7, 40985. 10.1038/srep40985 28106124 PMC5247688

[B111] Stockand J. D. Meszaros J. G. (2003). Aldosterone stimulates proliferation of cardiac fibroblasts by activating Ki-RasA and MAPK1/2 signaling. Am. J. Physiol. Heart Circ. Physiol. 284, H176–H184. 10.1152/ajpheart.00421.2002 12388314

[B112] Sun M. Chen M. Dawood F. Zurawska U. Li J. Y. Parker T. (2007). Tumor necrosis factor-alpha mediates cardiac remodeling and ventricular dysfunction after pressure overload state. Circulation 115, 1398–1407. 10.1161/CIRCULATIONAHA.106.643585 17353445

[B113] Sun Y. Zhang J. Zhang J. Q. Weber K. T. (2001). Renin expression at sites of repair in the infarcted rat heart. J. Mol. Cell. Cardiol. 33, 995–1003. 10.1006/jmcc.2001.1365 11343421

[B114] Takahashi K. Sakamoto K. Kimura J. (2012). Hypoxic stress induces transient receptor potential melastatin 2 (TRPM2) channel expression in adult rat cardiac fibroblasts. J. Pharmacol. Sci. 118, 186–197. 10.1254/jphs.11128fp 22293297

[B115] Talman V. Ruskoaho H. (2016). Cardiac fibrosis in myocardial infarction-from repair and remodeling to regeneration. Cell Tissue Res. 365, 563–581. 10.1007/s00441-016-2431-9 27324127 PMC5010608

[B116] Tarbit E. Singh I. Peart J. N. Rose'meyer R. B. (2019). Biomarkers for the identification of cardiac fibroblast and myofibroblast cells. Heart fail. Rev. 24, 1–15. 10.1007/s10741-018-9720-1 29987445

[B117] Um J. Y. Kang S. Y. Kim H. J. Chung B. Y. Park C. W. Kim H. O. (2020). Transient receptor potential vanilloid-3 (TRPV3) channel induces dermal fibrosis via the TRPV3/TSLP/Smad2/3 pathways in dermal fibroblasts. J. Dermatol. Sci. 97, 117–124. 10.1016/j.jdermsci.2019.12.011 31959383

[B118] Umbarkar P. Ejantkar S. Tousif S. Lal H. (2021). Mechanisms of fibroblast activation and myocardial fibrosis: Lessons learned from FB-Specific conditional mouse models. Cells 10, 2412. 10.3390/cells10092412 34572061 PMC8471002

[B119] Vallee A. Lecarpentier Y. (2019). TGF-beta in fibrosis by acting as a conductor for contractile properties of myofibroblasts. Cell Biosci. 9, 98. 10.1186/s13578-019-0362-3 31827764 PMC6902440

[B120] Villalobos E. Criollo A. Schiattarella G. G. Altamirano F. French K. M. May H. I. (2019). Fibroblast primary cilia are required for cardiac fibrosis. Circulation 139, 2342–2357. 10.1161/CIRCULATIONAHA.117.028752 30818997 PMC6517085

[B121] Voets T. Nilius B. Hoefs S. Van Der Kemp A. W. Droogmans G. Bindels R. J. (2004). TRPM6 forms the Mg^2+^ influx channel involved in intestinal and renal Mg^2+^ absorption. J. Biol. Chem. 279, 19–25. 10.1074/jbc.M311201200 14576148

[B122] Wang Q. Ma S. Li D. Zhang Y. Tang B. Qiu C. (2014). Dietary capsaicin ameliorates pressure overload-induced cardiac hypertrophy and fibrosis through the transient receptor potential vanilloid type 1. Am. J. Hypertens. 27, 1521–1529. 10.1093/ajh/hpu068 24858305

[B123] Wang Q. Zhang Y. Li D. Zhang Y. Tang B. Li G. (2016). Transgenic overexpression of transient receptor potential vanilloid subtype 1 attenuates isoproterenol-induced myocardial fibrosis in mice. Int. J. Mol. Med. 38, 601–609. 10.3892/ijmm.2016.2648 27314441

[B124] Weber K. T. Sun Y. Bhattacharya S. K. Ahokas R. A. Gerling I. C. (2013). Myofibroblast-mediated mechanisms of pathological remodelling of the heart. Nat. Rev. Cardiol. 10, 15–26. 10.1038/nrcardio.2012.158 23207731

[B125] Weber K. T. Sun Y. Katwa L. C. (1997). Myofibroblasts and local angiotensin II in rat cardiac tissue repair. Int. J. Biochem. Cell Biol. 29, 31–42. 10.1016/s1357-2725(96)00116-1 9076939

[B126] Wu Y. Liu Y. Pan Y. Lu C. Xu H. Wang X. (2018). MicroRNA-135a inhibits cardiac fibrosis induced by isoproterenol via TRPM7 channel. Biomed. Pharmacother. 104, 252–260. 10.1016/j.biopha.2018.04.157 29775892

[B127] Yoshie K. Rajendran P. S. Massoud L. Mistry J. Swid M. A. Wu X. (2020). Cardiac TRPV1 afferent signaling promotes arrhythmogenic ventricular remodeling after myocardial infarction. JCI Insight 5, 124477. 10.1172/jci.insight.124477 31846438 PMC7098788

[B128] Yousefi F. Shabaninejad Z. Vakili S. Derakhshan M. Movahedpour A. Dabiri H. (2020). TGF-Beta and WNT signaling pathways in cardiac fibrosis: Non-coding RNAs come into focus. Cell Commun. Signal. 18, 87. 10.1186/s12964-020-00555-4 32517807 PMC7281690

[B129] Yu N. Jiang J. Yu Y. Li H. Huang X. Ma Y. (2014). SLC41A1 knockdown inhibits angiotensin II-induced cardiac fibrosis by preventing Mg^2+^ efflux and Ca^2+^ signaling in cardiac fibroblasts. Arch. Biochem. Biophys. 564, 74–82. 10.1016/j.abb.2014.09.013 25263961

[B130] Yu Y. Chen S. Xiao C. Jia Y. Guo J. Jiang J. (2014). TRPM7 is involved in angiotensin II induced cardiac fibrosis development by mediating calcium and magnesium influx. Cell Calcium 55, 252–260. 10.1016/j.ceca.2014.02.019 24680379

[B131] Yue L. Xu H. (2021). TRP channels in health and disease at a glance. J. Cell Sci. 134, jcs258372. 10.1242/jcs.258372 34254641 PMC8358089

[B132] Zhang Y. H. Sun H. Y. Chen K. H. Du X. L. Liu B. Cheng L. C. (2012). Evidence for functional expression of TRPM7 channels in human atrial myocytes. Basic Res. Cardiol. 107, 282. 10.1007/s00395-012-0282-4 22802050 PMC3442166

[B133] Zhong B. Rubinstein J. Ma S. Wang D. H. (2018). Genetic ablation of TRPV1 exacerbates pressure overload-induced cardiac hypertrophy. Biomed. Pharmacother. 99, 261–270. 10.1016/j.biopha.2018.01.065 29334670

[B134] Zhong H. Wang T. Lian G. Xu C. Wang H. Xie L. (2018). TRPM7 regulates angiotensin II-induced sinoatrial node fibrosis in sick sinus syndrome rats by mediating Smad signaling. Heart Vessels 33, 1094–1105. 10.1007/s00380-018-1146-0 29511803 PMC6096742

[B135] Zhou Y. Yi X. Wang T. Li M. (2015). Effects of angiotensin II on transient receptor potential melastatin 7 channel function in cardiac fibroblasts. Exp. Ther. Med. 9, 2008–2012. 10.3892/etm.2015.2362 26136930 PMC4471700

